# Effects of Social Implementation Education for Assistive Device Engineers at NIT (KOSEN) through the Development of a Digital Reading Device for the Visually Impaired

**DOI:** 10.3390/s22031047

**Published:** 2022-01-28

**Authors:** Kimiyasu Kiyota, Takaaki Ishibashi, Manabu Shimakawa, Kazuyuki Ito

**Affiliations:** 1 Board of Administration, National Institute of Technology (KOSEN), Kumamoto College, Koshi-City 861-1102, Japan; Shimakawa@kumamoto-nct.ac.jp; 2 Faculty of Electronics and Information Systems Engineering, National Institute of Technology (KOSEN), Kumamoto College, Koshi-City 861-1102, Japan; ishibashi@kumamoto-nct.ac.jp; 3 National Rehabilitation Center for Persons with Disabilities, 4820-1, Imazu, Nishi-ku, Fukuoka-City 819-0165, Japan; kazuyukiw650@gmail.com

**Keywords:** assistive technology, visually impaired support, education of engineers, text reading

## Abstract

Assistive technology (AT) is any item, device, software, or product system used to enhance, maintain, or improve the functional abilities of people with disabilities. There are many people with disabilities in the world, including the visually impaired, the hearing impaired, and the physically impaired. We established the National KOSEN Support Equipment Development Network (KOSEN-AT) with technical college faculty members 10 years ago to assist these disabled and elderly people. However, Japan is facing the challenge of a rapidly aging society, and the digital transformation of assistive device development for people with disabilities has not been adequately addressed. A major reason for this is the lack of engineers in Japan who can develop products with an understanding of the needs of people with disabilities and the elderly. In this paper, we describe a new initiative of the GEAR 5.0 program, a practical engineer education program that will enable the development of assistive devices for the physically challenged and the elderly, which started in 2020 at the National Institute of Technology in Japan. We believe that it is necessary to educate technicians not only with conventional specialized skills, but also with a full understanding of the concept of disability and basic skills in assistive technology. Next, we developed “Touch Talker”, a digital text reading system for the visually impaired. As a part of the GEAR 5.0 program, we conducted an evaluation experiment in which students from a technical college experienced visual impairment in the same blindfolded environment as visually impaired people to evaluate the developed assistive device. To verify its importance, we developed a digital text-to-speech system for the visually impaired, “Touch Talker”, as part of the GEAR 5.0 program. We thought that by conducting evaluation experiments in the same blindfolded environment as visually impaired people, we could make technical college students aware of the difficulties of operating digital devices due to visual impairment. The results of the experiment showed that the developed “Touch Talker” was effective for both the visually impaired group and the blindfolded technical college student group. The evaluation results also showed a similar trend, confirming that the evaluation by blindfolded technical college students is effective for the development of assistive devices for the visually impaired. In addition, the technical college students who participated in the evaluation experiment were able to understand the difficulty of operating digital devices by experiencing visual impairment. It was suggested that the perspective of the people involved is important in the development of assistive devices.

## 1. Introduction

Recently, the enhancement of welfare and medical care for the super-aging society has become an issue not only in Japan but also on a global scale. The Japanese Ministry of Education, Culture, Sports, Science and Technology (MEXT) recently imposed an obligation on all employers to employ people with disabilities at a rate higher than the legal employment rate, to encourage “people with disabilities to live and work together as members of the community” [1]. In response to this social situation, the MEXT has taken the lead in promoting policies in various schools that promote the early realization of an inclusive society based on rational consideration [2,3]. Under these circumstances, the demand for AT devices for the disabled and elderly is increasing in the welfare equipment industry and educational institutions, and there is an urgent need to train engineers who can develop assistive devices.

National colleges of technology (NCTs) are higher education institutions that accept graduates of junior high schools and provide an integrated education for 5 years (5.5 years for merchant marine technical colleges) to train engineers required by society. Many engineers with specialties in mechanical, electrical, information, biochemical, architectural, and civil engineering have come from NCTs [4]. However, the development of AT devices necessitates not only traditional, specialized skills but also new technical skills (AT skills) that take into account people’s needs (disabled and elderly users). In other words, it is preferable to introduce technical education that employs a social implementation model (needs-based) for the development and evaluation of devices and orthotics in collaboration with specialists from various fields such as medical and welfare care. In response to such requests from special support schools and medical institutions in each prefecture, the faculty members of 13 NCTs took the lead in establishing the National KOSEN Assistive Technology (AT) Development Network (KOSEN-AT Net). In this network, each technical college has developed various devices and applications for special needs [5,6,7,8,9,10,11,12].

## 2. Enhancement of Technical College Education (AT Future Technology Human Resource Development Model for a Sustainable Society)

### 2.1. GEAR 5.0 Project and eAT (Extended Assistive Technology)

In Japan, the National Institute of Technology (KOSEN) started its latest research project, called GEAR 5.0, in May 2020. AT and medical engineering are key disciplines selected as the focus of the GEAR 5.0 project. This project aims to realize the next-generation AT (extended-AT (eAT)) that combines AT and digital technologies (AI, IoT, robotics, big data, and mobility, etc.) to realize Society 5.0. Here, “Society 5.0” refers to Japan’s 5th Science and Technology Basic Plan, which refers to an ultra-smart society that utilizes digital technologies such as IoT and AI that integrate cyber and physical space.

**Figure 1:**
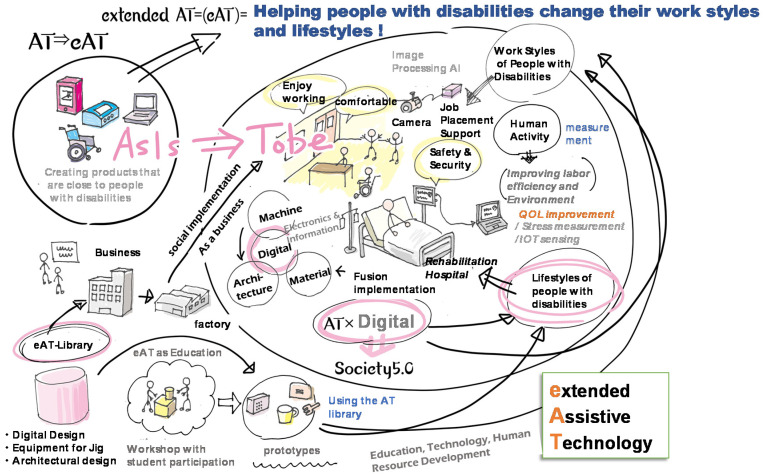
Conceptual diagram of the eAT (extended-AT) in the GEAR 5.0 project.

Figure 1 shows the conceptual idea of the eAT (extended-AT) project in GEAR 5.0. We will encourage market-creating research that will result in increased employment opportunities for people with disabilities, improved quality of life for patients and people with disabilities, and increased healthy life expectancy for the elderly. Simultaneously, students at KOSEN(NIT colleges) will participate in the process of social implementation, acquiring a practical knowledge of advanced technologies through problem-solving activities and the development of prototype devices and services, and developing as engineers with an AT mindset who can solve problems from the perspective of people with disabilities. To promote the above research and education, we will accumulate and share the know-how of the design data and customization of the next-generation ATs developed by the technical colleges participating in GEAR 5.0 as an AT library that can be shared by all technical colleges in Japan, to improve the development efficiency of AT devices and services and promote the social implementation of ATs. Furthermore, we will establish a joint research network with medical and welfare institutions, companies, and local governments, among others, to share the voices of AT users, on-site needs, and issues, and to jointly create better AT devices and services, as well as to build a research system for industry–academia–government collaboration. The goal of this project is to produce engineers from technical colleges across the country who can realize “the creation of a symbiotic society by using eAT even for people with disabilities” [13]. These pilot initiatives that incorporate new technologies to extend healthy life expectancy and improve wellbeing are at the beginning stages of consideration as a challenge not only for Japan but also for the world [14,15].

### 2.2. Enhancement of Technical College Education

**Figure 2:**
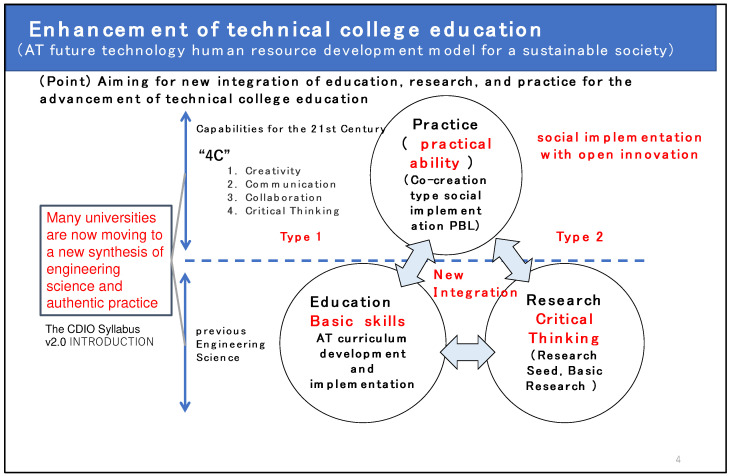
Conceptual diagram of the eAT (extended-AT) in GEAR 5.0 project.

Figure 2 shows the concept of the advancement of technical college engineer education in this GEAR 5.0 project (nursing care and medical engineering fields). In the field of AT, we aim for a new integration of education, research, and practice for the development of technical college education. In other words, we will practice the fusion of education, research, and social implementation that is being promoted at many universities. AT has so far focused on the production of assistive devices to overcome or complement disabilities. In the next generation, however, it will be essential to have a good understanding of people with disabilities when developing high-mix, low-volume assistive devices for various disabilities using digital technology. Therefore, this is a gap market where no major companies have been able to enter. Until now, the technical colleges collaborating in the nationwide KOSEN-AT network have been working individually with local special-needs schools, NPOs in Japan, and medical institutions to develop and research assistive devices.

## 3. New Concept of the “Touch Talker” System

### 3.1. Overview and Background of the Study

People with disabilities who suddenly lose their sight due to diseases or traffic accidents cannot easily read documents using digital terminals. In recent years, tablets for the visually impaired have been developed. BLITAB allows users to read entire pages on a tactile screen. BLITAB enables blind users for the first time to learn, work, and play on a single mobile device, providing digital access to information in real time. BLITAB converts any document into braille text. However, this system is for users who have access to braille [16].

The following tablet terminal applications have been developed for use by the blind and elderly who cannot use braille. AccessNote for the iOS platform is designed particularly for VoiceOver users looking for an efficient, feature-rich note-taking experience. An inexpensive alternative to traditional note-takers, it allows users to combine efficient note-taking with the other features and functions of the iOS devices, making it accessible to blind and visually impaired people [17]. AccessWorld has also been optimized for iOS VoiceOver and accessibility features. Amazon Kindle allows users to use their iPhones and iPads as a Kindle device and access their e-books purchased from Amazon as well as download e-books directly to their devices [17]. However, with Amazon Kindle, visually impaired users have a hard time finding the part they want to read because the text is read aloud from the beginning of the document. The environment for ICT-assisted reading support described above is not sufficient for the visually impaired, especially those with intermediate visual impairment. For this reason, there is an urgent need to develop assistive devices based on Society 5.0 that can be used with an understanding of the situation of users with disabilities. Especially for the visually impaired, retrieving the necessary information from a vast amount of information is a very important task for users who listen from voice output devices.

**Figure 3:**
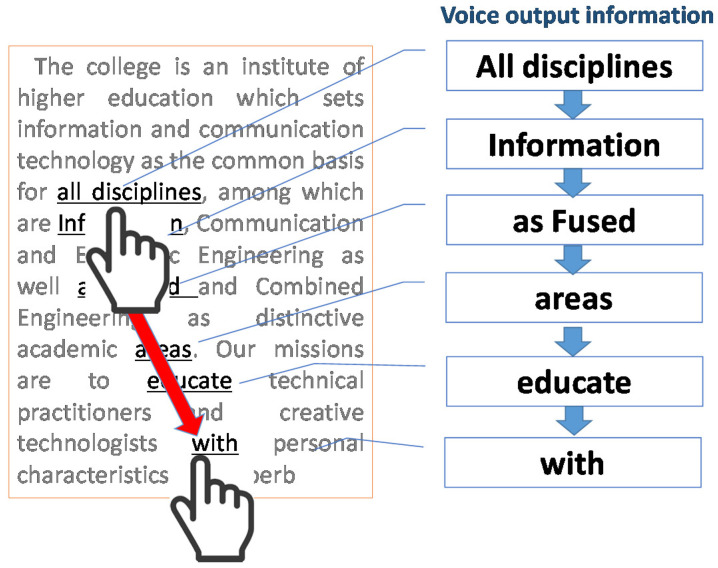
Conceptual diagram of text-to-speech using “finger tracing”.

The goal of this research is to create a digital smart terminal that allows visually impaired people to read tactilely by “finger tracing” the freely displayed text characters without any visual feedback. To realize this assistive technology, we propose a new system (dubbed “Touch Talker”) that outputs text at a specific position from a large number of text documents displayed on a smart terminal’s screen by “tracing” the screen with a finger as shown in Figure 3.

The number of visually impaired people in Japan is approximately 316,000 (Ministry of Health, Labor and Welfare, 2013). Many of these visually impaired people are pursuing specialized education in physical therapy courses at national facilities and schools for the blind, to become professionally self-sufficient by obtaining national qualifications such as acupuncture and moxibustion therapists. However, only 9.2% of the blind use braille, and 50% of the blind do not use writing instruments. The use of text-to-speech software, which reads out text information on the screen, has improved the PC usage environment for the visually impaired; however, the PC usage rate for general braille users is only 10.7%, and the rate for the visually impaired remains around 5% (about 15,000 people). In addition, the rapid spread of smart terminals in recent years has created a new digital divide for the visually impaired, as they are unable to use soft key operations on the screen. To improve these problems, we thought that we could provide employment opportunities for the visually impaired by making the effective use of smart terminals possible for them.

From our previous research, we have developed a pen input system for the visually impaired that allows them to input Japanese into a computer using the same method as their usual writing (Fundamental Research (C) Representative: Kiyota: FY 2007–2008). In our previous research, we have commercialized a pen-based Japanese character input system for Japanese document processing. This research is an effort to demonstrate a means of obtaining text information at high speed using a digital terminal in order to solve the digital divide for the visually impaired.

### 3.2. Development Environment

For the development of the “Touch Talker” system, we used a development environment called Xcode (Apple’s Integrated Development Environment (IDE) for software development). We also used Swift, a programming language provided by Apple for developing iOS applications. As a voice output for the function of reading out text and PDF documents from a computer, we used a speech synthesizer called “VoiceOver” (Apple’s proprietary VoiceOver technology), which is compatible with iOS. When importing text documents, the Japanese morphological analysis software “MeCab” was used for pre-processing separate sentences into parts of speech and to convert sentences around instructions into word units for tracing and reading.

(a) Xcode
  Xcode is Apple’s Integrated Development Environment (IDE) for developing applications for iOS.
(b) Swift
  Swift is a robust and intuitive programming language created by Apple to developapplications for iOS, Mac, Apple TV, and Apple Watch.
(c) MeCab
  Morphological analysis is an analysis that decomposes a sentence into morphemes (the smallest unit in which a word has meaning) based on the grammar of the target language and part-of-speech information of the word. We have used the free MeCab software for the morphological analysis in this study. “Touch Talker” uses the above software to perform morphological analysis on all documents at the time of loading text files, and separates words on the basis of parts of speech. We also set it up to read out the word closest to the coordinate position of the finger tap.

### 3.3. Specifications

**Figure 4:**
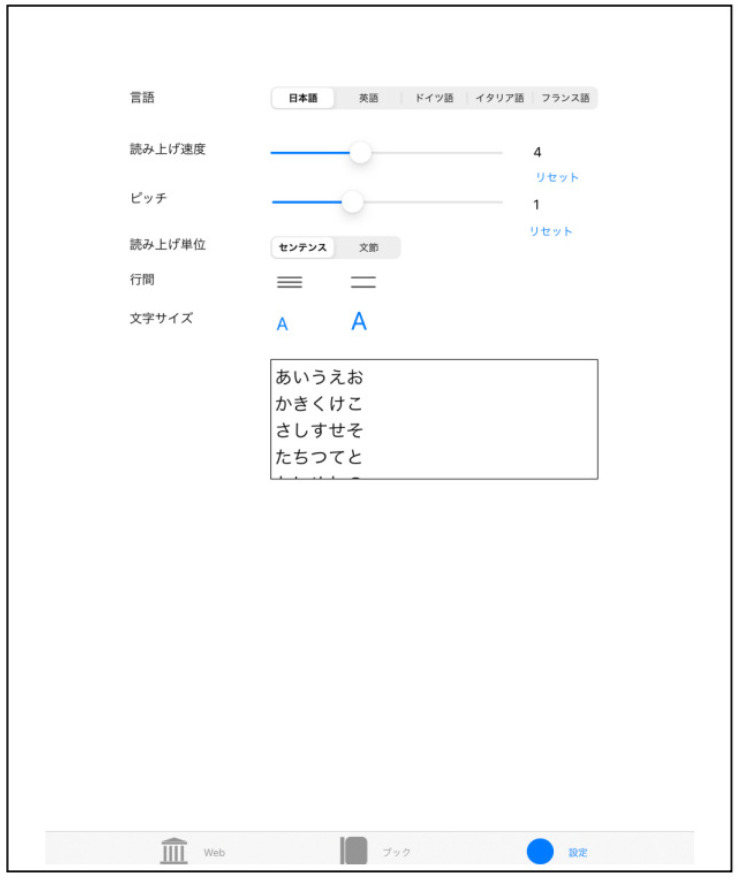
Screenshot of settings and contents in “Touch Talker”. The example screen in Figure 4 shows the Touch Talker settings menu. From the top, it shows “Language Selection”, “Reading Speed”, “Pitch (character spacing)”, “Reading Unit–Sentence or Section”, and “Font Size”. In addition, the rectangle at the bottom is an example of a sentence display.

**Figure 5:**
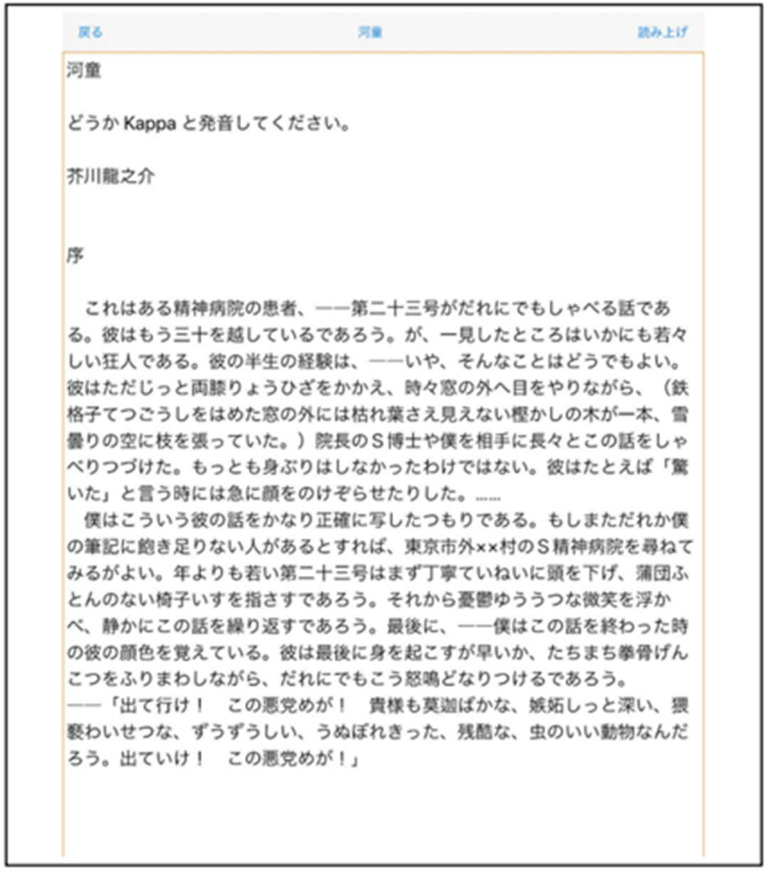
View the text document of a file in Japanese. The Japanese text in Figure 5 is a part of a novel called “Kappa” written by a Japanese author named Ryunosuke Akutagawa.

Using a smart device, start the “Trace Reading” application. Trace the file list in the text folder from top to bottom, and VoiceOver will read out the individual file titles. Next, open the text document that you want to read by selecting the text file you want to read. Begin reading by tracing from above with your index finger. The text document you want to read can also be added or deleted as a text file or PDF file from “iTunes” or “folder”. Table 1 and Figure 4 show the list of settings and screenshots for “Touch Talker”. In the setting mode, the user can set each language (Japanese, English, German, Italian, French), reading speed, pitch, reading units, (sentences, clauses), line spacing, font size, etc., according to the user’s preference. Depending on the size of the user’s hands and their senses, there are different settings for font size and line spacing according to their preference. This system uses text-to-speech software to read out the settings and allow the user to change the settings, but in this case, we used the default settings that the subjects had practiced with before the experiment, and set them all to the same settings. Here, I used a font size of 28 points, a line spacing of 5 points, and a reading speed of 5 (middle speed). Figure 5 shows a screenshot of the loaded text document. After booting up the system, the user uses their index finger to trace down from the top of the screen to read the word closest to their fingertip. When the user’s index finger reaches the bottom of the screen, a double-tap with the index and middle fingers brings up the next page (this is a type of gesture control).

## 4. Functionality Evaluation Experiment

### 4.1. Experiment Participants

We conducted an evaluation experiment to verify the effectiveness of our proposed system with word-reading function by finger tracing. The experiment required two groups of six visually impaired people and six blindfolded healthy people to participate. Table 2 shows the profiles of the six visually impaired people, and Table 3 shows the profiles of six healthy, blindfolded people (NIT, Kumamoto college students). As a result, as part of their social implementation education, these students were allowed to experience the difficulties of people with disabilities. Table 2 shows the participants’ visual impairment status as well as the digital devices they typically use. Furthermore, we asked subjects of a young age to participate to avoid the effects of limb disability.

In general, visually impaired people obtain information by using digital IC recorders (hereinafter referred to as “IC.R”) for voice playback, and by using text-to-speech software on PCs and tablet terminals.

### 4.2. Comparison Experiment with the Information Search Task

**Figure 6:**
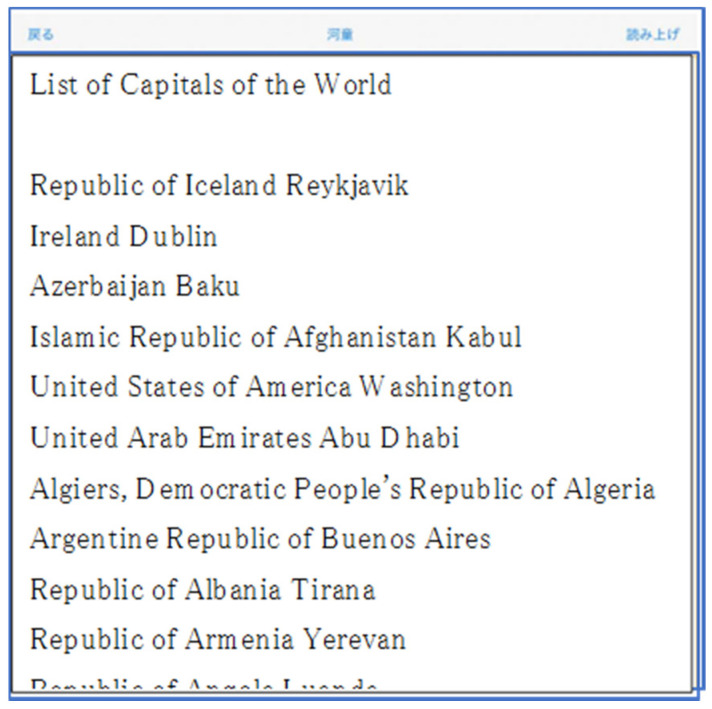
A part of the text file (6 pages) of the list of names and capitals of the countries of the world (190 countries) was prepared for the evaluation experiment.

For the evaluation experiment, we prepared a task of retrieving arbitrary information from a huge amount of information. As shown in Figure 6, we also created a text file with 190 countries and their capitals, one on each line. The prepared text file was then loaded into the developed “Touch Talker” system, as shown in Figure 6. In this experiment, the specifications of the initial system settings in Table 1 were set as follows:

/Language: Japanese
/Reading speed: 4 steps (middle speed)
/Pitch 1.0 (character spacing)
/Reading units: phrase
/Line space: 14 points
/Font size: 14 points

With the speech settings described above, the names of all 190 countries and their capitals were read out loud using the VoiceOver function of the iPad from the top to the end of the text and recorded on a digital IC recorder.

### 4.3. Information Search Task

We obtained informed consent from each subject before they participated in the experiment. In the evaluation experiment, the experimenter first explained how to operate the digital IC.R (Digital IC recorder) and the iPad. Then, the subjects were asked to practice the operation method for about 10 min.

To test the finger tracing function’s effectiveness, we first loaded text data into “Touch Talker” and recorded the same voice data read out at the same speed on a digital recording device. The subjects were instructed to locate the capital of the country using “Touch Talker” and the IC.R. In this study, we created a six-page textbook for evaluation that lists the world’s countries (190 countries) and capitals alphabetically at one-line intervals, as shown in Figure 6.

**Figure 7:**
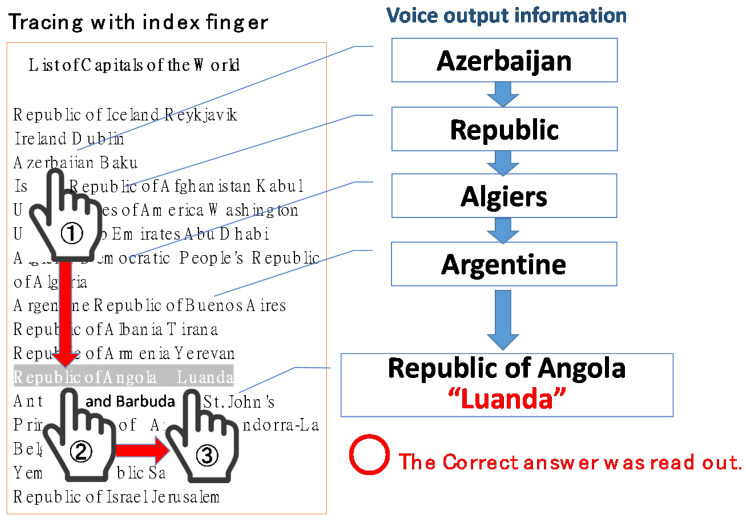
List of capitals of 190 countries used in the evaluation experiments.

Figure 7 shows the flow of capital search by tracing with your index finger. Using a smart device, launch the “Trace and Read” application. When the user traces their index finger down from the top of the iPad screen, the name of the country at their fingertip will be read out by the iPad’s VoiceOver function. Then, moving the user’s finger to the right will read out the capital of the country, as shown in Figure 7.

/Tasks for the evaluation experiment

**[First Time Trial]**

For each question, the experimenter read out the target country. Then, the measurement was started and the time taken by the subject to find the capital of the country was measured.

1st time: Measurement of the mean search time for a total of 10 countries

**Question 1**: Find the names of the capitals of the following countries.

**Task 1** “Touch Talker” → IC.R (Digital IC recorder) (5 countries)

(1). United Arab Emirates → **Abu Dhabi**
(2). Canada → **Ottawa**
(3). Grenada → **St. George’s**
(4). Kingdom of Saudi Arabia → **Riyadh**
(5). Czech Republic → **Prague**
  **Task 2** IC.R (Digital IC recorder) → “Touch Talker” (5 countries)
(6). State of Israel → **Jerusalem**
(7). Republic of Estonia → **Tallinn**
(8). Republic of Cameroon → **Yaounde**
(9). Solomon Islands → **Honiara**
(10). Republic of Chile → **Santiago**

**[Second Time Trial]**

The second trial was completed the same as the first time, with the experimenter reading out the target country. Then, the measurement was started and the time taken by the subject to find the capital of the country was measured.

2nd time: Measurement of the mean search time for a total of 10 countries.

**Question 2**: Find the name of the capital of the following countries.

**Task 3** (“Touch Talker” only)

(1). United Arab Emirates → **Abu Dhabi**
(2). Canada → **Ottawa**
(3). Grenada → **St. George’s**
(4). Kingdom of Saudi Arabia → **Riyadh**
(5). Czech Republic → **Prague**
(6). Israel → **Jerusalem**
(7). Estonia → **Tallinn**
(8). Republic of Cameroon → **Yaounde**
(9). Solomon Islands → **Honiara**
(10). Chile → **Santiago**

### 4.4. Experimental Results

**Figure 8:**
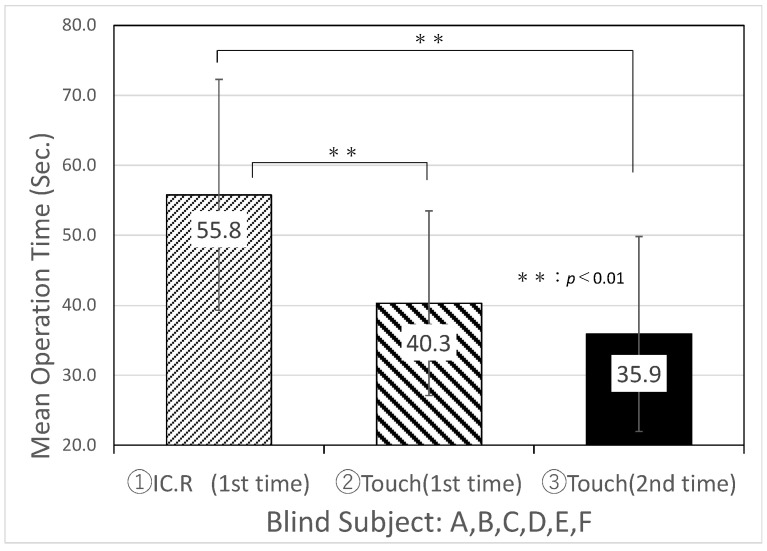
Results of an experiment comparing information retrieval between IC.R (Digital IC recorder) and “Touch Talker” by six visually impaired persons.

**Figure 9:**
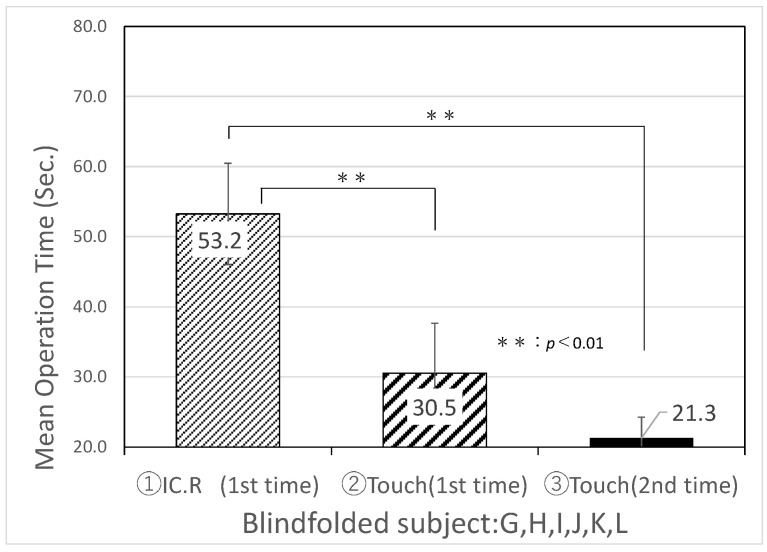
Results of an experiment comparing information retrieval between IC.R (digital IC recorder) and “Touch Talker” by blindfolding six healthy persons.

Table 4 and Figure 8 show the experimental results of the average search time and standard deviation of IC.R and “Touch Talker” for the first trial of Task 1, Task 2, and the second trial of Task 3 by six visually impaired people. Table 5 and Figure 9 show the experimental results of the mean search time and standard deviation for six blindfolded technical college students simulating intermediate visual impairment. From the comparison of Table 4 and Table 5, there was no significant difference in the mean search time using the digital IC recorder between the visually impaired group and the blindfolded group of students, which were recorded as 55.8 and 53.2 s, respectively (a p-value less than 0.01 s was considered statistically significant). The developed “Touch Talker”, on the other hand, reduced the mean search time for both the visually impaired people and blindfolded, healthy people when compared to the digital IC recorder. Furthermore, when compared to the first trials of Task 1 and Task 2, the average search time for both groups of “Touch Talker” became shorter in Task 3, the second trial, as they became more familiar with the operation of the device. According to the results of this experiment, the standard deviation for each individual decreased.

## 5. Discussion

Although the subjects were relatively the same age in this evaluation experiment, the average search time of the healthy group of technical college students who usually use smartphones and PCs became faster in the second trial of Task 3. This is thought to be due to the user’s familiarity with the operation of the iPad on which “Touch Talker” is installed. However, there was no difference in the retrieval time of the first trial for subject B in the visually impaired group when using either “Touch Talker” or the digital IC recorder.

**Figure 10:**
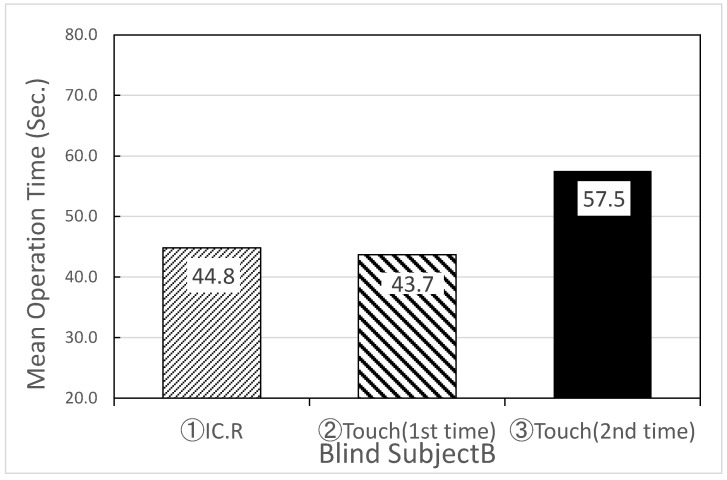
Results of the capital search experiment for subject B with visual impairment.

Rather, the second trial of “Touch Talker” tended to take more time as shown in Figure 10. Subject B is a 30-year-old blind female, as shown in Table 2. She usually uses most of the information terminals, including a smartphone. Therefore, she is familiar with information terminals. In the questionnaire after the experiment, she commented on the operation of “Touch Talker” as follows:

“I couldn’t tell whether my finger was on a letter, a space, or a blank line.”

“In many cases, I could not read the text even if my finger was on the text, so I just kept moving my finger.”

From her comments, we think that the main reason for the unresponsive state is due to the poor response of tapping on the iPad, and we would like to improve our next application.

On the other hand, the participating technical college students said that operating the tablet device while blindfolded was completely different from their normal work and that they had a hard time just operating the touch screen. From this evaluation experiment, it was found that even for technical college students who are used to operating tablets, when blindfolded, there was a tendency to operate the tablet in a similar way as the visually impaired. Furthermore, we were able to make the technical college students understand the importance of considering the situation of the subject as an engineer when developing assistive devices, and we confirmed the effectiveness of social implementation education.

## 6. Conclusions

In this paper, we described the necessity of the next-generation AT (extended-AT, eAT thereafter) that integrates AT (Assistive Technology) and digital technologies (AI, IoT, robotics, big data, mobility, etc.) for the realization of Society 5.0. Furthermore, we described GEAR 5.0, a project to train the next generation of engineers to support people with disabilities through technological measures, to expand employment opportunities for people with disabilities, and to improve the quality of life of patients and people with disabilities. Next, we created “Touch Talker” to help visually impaired people read text using a digital terminal, and we described an evaluation experiment with visually impaired people conducted by technical college students using the prototype. We asked the technical college students to simulate visual impairment by performing the same task as the visually impaired while blindfolded in the evaluation experiment.

From the evaluation experiments, we confirmed the effectiveness of our proposed “Touch Talker”, which reads out text traced with the index finger. However, we discovered some flaws in the application, such as the tap control function. We intend to improve the application and turn it into a commercial device that can be used by visually impaired people based on the information obtained from these evaluation experiments and questionnaires.

## 7. Patents

Part of this study has been granted Japan patent JP6391064B, “Audio output processing device, audio output processing program, and audio output processing method”, Japan, 31 August 2018. Additionally, “Touch Talker” is registered as a trademark under JP6368537, 25 March 2021.

## Figures and Tables

**Table 1:** Settings and contents for “Touch Talker”.

Setting Item	Contents	Setting Range
Language	Select language	Japanese, English, German, Italian, French
Reading speed	Change the reading speed	1–9 steps
Pitch	Change the pitch of the voice	0.5–2.0
Reading units	Change the unit of reading	Sentence, phrase
Line space	Set line spacing	0–39 point
Font size	Set font size	20–40 point

**Table 2:** Profile of the six visually impaired subjects that participated in the experiment.

Subject	A	B	C	D	E	F
Visual impairment	Amblyopia and narrow vision	Total blindness	Total blindness	Amblyopia and narrow vision	Total blindness	Total blindness
Gender	Male	Female	Male	Female	Male	Female
Ages	21	30	38	26	32	24
Duration of disability	4 years	30 years	5 years	12 years	5 years	4 years
Usual information methods	Smartphone	All except “Galapagos” cell phone	PC, smartphone, braille	Cell phone, radio, TV, iPad	“Galapagos” cell phone, PC, braille, Daisy	Smartphones, braille, Daisy, TV

**Table 3:** Profiles of the six blindfolded, healthy subjects that participated in the experiment.

Subject	G	H	I	J	K	L
Visual impairment	No visual impairment, blindfolded	No visual impairment, blindfolded	No visual impairment, blindfolded	No visual impairment, blindfolded	No visual impairment, blindfolded	No visual impairment, blindfolded
Gender	Female	Female	Male	Male	Female	Male
Ages	19	20	20	20	15	19
Duration of disability	--	--	--	--	--	--
Usual information methods	Smartphone, PC	Smartphone, PC	Smartphone, PC	Smartphone, PC	Smartphone, PC	Smartphone, PC

**Table 4:** Results of an experiment comparing information retrieval between IC.R (Digital IC recorder) and “Touch Talker” by a visually impaired person.

Task	Avg. (S)	SD (S)
① IC.R (1st time)	55.8	16.5
② Touch (1st time)	40.3	13.2
③ Touch (2nd time)	35.9	13.9

**Table 5:** Results of an experiment comparing information retrieval between IC.R (Digital IC recorder) and “Touch Talker” by blindfolding a healthy person.

Task	Avg. (S)	SD (S)
① IC.R (1st time)	53.2	7.2
② Touch (1st time)	30.5	7.1
③ Touch (2nd time)	21.3	3.0

## Data Availability

Not Applicable.
